# The Potential of *Cistus salviifolius* L. to Phytostabilize *Gossan* Mine Wastes Amended with Ash and Organic Residues

**DOI:** 10.3390/plants11050588

**Published:** 2022-02-22

**Authors:** Luísa C. Carvalho, Erika S. Santos, Jorge A. Saraiva, M. Clara F. Magalhães, Felipe Macías, Maria Manuela Abreu

**Affiliations:** 1 Linking Landscape, Environment, Agriculture and Food Research Center, Associated Laboratory TERRA, Instituto Superior de Agronomia, Universidade de Lisboa, 1349-017 Lisboa, Portugal; erikasantos@isa.ulisboa.pt (E.S.S.); mclara@ua.pt (M.C.F.M.); manuelaabreu@isa.ulisboa.pt (M.M.A.); 2 QOPNA & LAQV-REQUIMTE, Departamento de Química, Campus Universitário de Santiago, Universidade de Aveiro, 3810-193 Aveiro, Portugal; jorgesaraiva@ua.pt; 3 School of Biological, Earth & Environmental Sciences, UNSW Sydney, Sydney, NSW 2052, Australia; 4 Departamento de Edafología y Química Agrícola, Facultad de Biología, Campus Universitario Sur, Universidad de Santiago de Compostela, 15782 Santiago de Compostela, Spain; felipe.macias.vazquez@usc.es; 5 Instituto de Investigaciones Tecnológicas, Campus Universitario Sur, Universidad de Santiago de Compostela, 15782 Santiago de Compostela, Spain

**Keywords:** bioaccumulation, oxidative stress, PHE, soil amendments, Technosols

## Abstract

The São Domingos mine is within the Iberian Pyrite Belt, a mining district with large concentrations of polymetallic massive sulfide deposits. Mine waste heaps are considered extreme environments, since they contain high total concentrations of potentially hazardous elements (PHE), which contribute to inhibiting the development of most plants. Autochthonous plant species, such as *Cistus salviifolius* L., are able to grow naturally in this degraded environment, and may contribute to minimizing the negative chemical impacts and improving the landscape quality. However, the environmental rehabilitation processes associated with the development of these plants (phytostabilization) are very slow, so the use of materials/wastes to improve some physicochemical properties of the matrix is necessary in order to speed up the process. This work studied the effectiveness of the phytostabilization with *C. salviifolius* of *gossan* mine wastes from the mine of São Domingos amended with organic and inorganic wastes in order to construct Technosols. The mine wastes have an acid pH (≈3.5), high total concentrations of PHE and low concentrations of organic C and available nutrients. The best vegetative development occurred without visible signs of toxicity in the Technosols containing a mixture of agriculture residues. These treatments allowed the improvement of the soil-plant system providing a better plant cover and improved several chemical properties of mine wastes, helping to speed up the environmental rehabilitation.

## 1. Introduction

The Iberian Pyrite Belt (IPB) is one of the most important volcanogenic massive sulfide ore deposits in the world [1], and it is located in the southwest of the Iberian Peninsula. It runs from WNW-ESE occupying an area of *circa* 250 by 40 km [1,2]. The IPB extends from the Atlantic coast of Portugal (Alentejo region) to the Spanish province of Seville and is characterised by high contents of metallic sulfide deposits [2,3]. At the end of the 20th century, after intensive mining activity during the 19th and 20th centuries, a large number of mines were closed and abandoned [3,4].

In the IPB mines, as a result of open cast and underground mining operations, large amounts of waste materials with high contents of potentially hazardous elements (PHE) have been exposed to weathering and pedogenesis conditions leading, in some areas, to the formation of incipient soils (Toxic Spolic Technosols, [5]). These soils, as well as the mine wastes, have substantial chemical and physical limitations, such as low organic matter and nutrient contents in the available fraction and low pH, unfavourable texture and structure and high total content of PHE [6]. Also, the oxidation of the metallic sulfides included in some mine spoils results in acid mine drainage (AMD) with the consequent release and leaching of significant amounts of PHE leading to the contamination of the surrounding soils and the alteration or destruction of the adjacent ecosystems [7,8,9]. These extreme conditions of soils/mine wastes can inhibit the growth of vegetation and reduce the vegetation cover, contributing to the increase of erosion and, consequently, to serious contamination in the surrounding areas [6]. The transfer of chemical elements among the different solid phases contributes to controlling their behavior, mobility and availability. 

Currently, in Portugal, there are no efficient and adequate maintenance programs for the environmental restoration of degraded ancient mining areas. However, some sustainable development policies have been defined, and they involve mineral extraction, prospecting for metal deposits, the environmental recovery of mining areas affected by acid mine drainage and the promotion of geologically and mining-focused thematic tourism [2]. The latter had a significant boost with the development of the Route of Mines and Points of Mining and Geological Interest in Portugal, of which the mine of São Domingos is a highlight [http://www.roteirodeminas.pt/route.aspx?v=b9536855-2506-49c8-8700-8651914b19e7 (accessed on 15 January 2022)].

Although mining areas present adverse characteristics for biodiversity, some autochthonous plant species, such as those of the *Cistus* genus, are well adapted to extreme environments and are able to grow naturally in these degraded environments without apparent symptoms of toxicity, contributing to minimize the negative chemical impacts and improving the landscape quality [10,11,12]. In fact, *Cistus salviifolius* L. is a species that grows spontaneously in several mining areas in the IPB, including São Domingos, as well as in uncontaminated areas near the mines [10,11]. This woody shrub is autochthonous of the Mediterranean basin and is also well adapted to the typical summer drought of the region [13] and to acid soils [14]. 

The environmental rehabilitation processes associated to the in situ development of autochthonous plants (phytostabilization) are very slow; therefore, the combined use of materials/wastes that improve some physicochemical properties of the matrix is necessary [15]. Moreover, in order to achieve the goals set by the 2030 Agenda for Sustainable Development regarding food safety, it is necessary to create effective and low-cost remediation technologies in areas suffering extensive soil contamination with PHE, and the former mines of the IPB are among those areas. This work studied the effectiveness of the phytostabilization with *C. salviifolius* of *gossan* mine wastes from the mine of São Domingos amended with organic/inorganic wastes to construct a Technosol. The amendments used for the elaboration of the Technosols were biomass ash, a mixture of agriculture residues (organic and inorganic wastes) and a mixture of both. This work evaluated the plants as well as *gossan* wastes and the constructed soils (Technosols elaborated with *gossan* wastes and organic/inorganic wastes) where they grew, comparing plant development and several physiological parameters related to stress response.

## 2. Results

### 2.1. Original Gossan Wastes and Analysis of the Amended Gossan Wastes 

*Gossan* is the result of the oxidation by weathering and leaching of sulfide deposits. The mineralogy of the *gossan* wastes from the São Domingos mine area is mainly composed of quartz, minor micas and typical mineral assemblages of sulfides oxidation, i.e., mainly iron oxides-hydroxides (hematite and goethite), jarosite, beudantite and Fe–Cu hydrated sulfates such as copiapite and poitevinite [16]. *Gossan* wastes have a very coarse texture, and, consequently, they do not pose any danger in air contamination by particulate material transfer. The original *gossan* wastes were amended with a mixture of organic/inorganic wastes from agriculture (OR) and biomass ashes (BA), an inorganic residue from a thermoelectric station, and a mixture of both (BA + OR). These mixtures produced different Technosols. The combination of the organic and inorganic correctives enables the assessment of which mixture complements a greater number of needs, both of the *gossan* wastes and of *C. salviifolius*, than applying the correctives separately.

The elemental concentrations in the total fraction of the original *gossan* wastes and of each organic and inorganic wastes that were used as amendments are shown on Table 1. The original *gossan* wastes had very high concentrations of several PHE, such as Cu, As and Pb, while all the amendments only showed trace amounts of those elements. 

**Figure 1:**
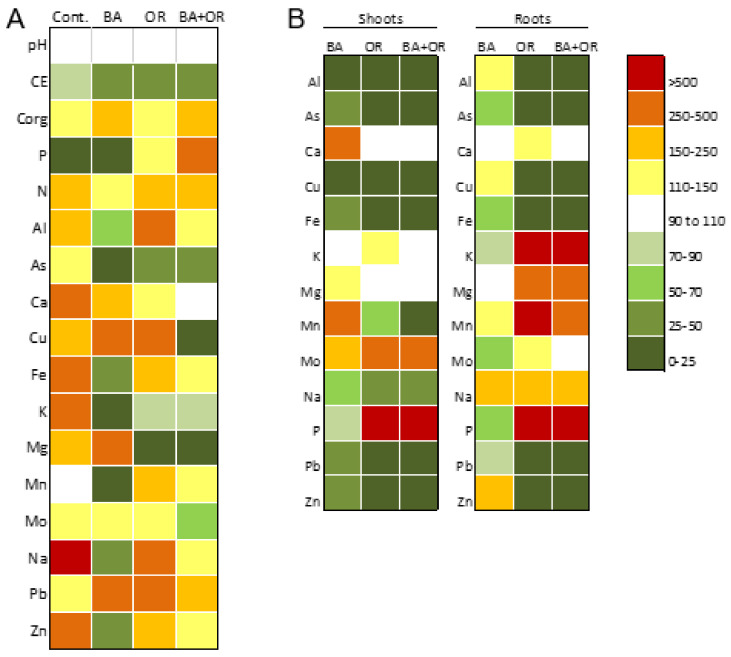
(**A**) Heat map showing the variation of general properties and elements concentration in the available fraction of the *gossan* wastes (control) and *gossan* amended (constructed soils-Technosols) with different wastes between the first sampling at one month incubation and the second sampling after one year of *Cistus salviifolius* growth. The variation is shown in percentage of change between the first sampling (considered 100%) and the second sampling. Individual values in Table 3 (first sampling) and Table 4 (second sampling). (**B**) Heat map showing the percentage of element accumulation in the shoots and roots of the three treatments (different Technosols) in relation to the unamended *gossan* wastes (control). The variation is shown in percentage of change between the control (considered 100%) and each of the Technosols. BA, biomass ash; OR, agricultural wastes.

The elemental composition in the available fraction, total N, extractable P and K, pH and electric conductivity (EC) of the *gossan* wastes and the Technosols in this assay, before and after the growth of *C. salviifolius* for one year, are shown on Table 2 and Table 3. The comparison between both conditions is expressed as a heat map in Figure 1. The application of the amendments, independently of the type, increased significantly both the pH and the EC of the originally very acid *gossan* wastes (control) (Table 2), while the growth of *Cistus* plants did not change pH significantly (Figure 1). Conversely, EC decreased significantly in all Technosols after plant growth, to similar values than the control, indicating an overall decrease in dissolved salts and availability of some elements (Figure 1). In Technosols where organic wastes were applied (OR and OR + BA), organic C, total N and extractable P increased by more than two fold in both samplings (Figure 1). 

Element availability in the Technosols changed with the amendments and with the plant growth. In the beginning of the assay, element concentrations in the available fraction of the Technosols increased from the control (untreated *gossan* wastes; with the exceptions of Cu, Mo and Pb), although increases were more evident in the Technosols containing a significant organic fraction (OR and OR + BA) (Table 3). The tendency for higher values in the Technosols with organic wastes was kept after one year of plant growth (with the exceptions of Cu, Mg and Pb). In this sampling, the differences between the control and the Technosol containing BA were the decrease of Cu and Pb availability while for As, Fe, Mo, Na, Pb and Zn, no significant differences were obtained (Table 4). The plant growth in *gossan* wastes (control) led to a general increase in the concentration of PHE in the available fraction. 

### 2.2. Element Distribution in Plants

After plant growth, the elemental composition of shoots and roots was quantified (Table 5), and the translocation factor (TF) was calculated (Table 6). In the shoots of plants grown in the *gossan* wastes, the concentration of many PHE and micronutrients was above the maximum limit considered as phytotoxic for plants in general, such as As and Cu in shoots (Table 5). Conversely, in the Technosols, only the single application of BA had levels of As, Mn and Pb above the maximum considered phytotoxic in shoots. In general, shoots of the control had higher concentrations of all elements than any of the Technosols, with the exception of K, P, Mg and Ca (Table 5) which had higher concentrations in the plants from Technosols with a single application of agricultural wastes (OR) (Table 5).

The tendency and intensity of element translocation from roots to shoots (translocation factor, TF) was modulated by element and amendments in the Technosols for almost all elements (Table 6). In general, similar behavior was obtained between the control and Technosols, with the exception of Mn and Zn. 

### 2.3. Plant Establishment and Growth

**Figure 2:**
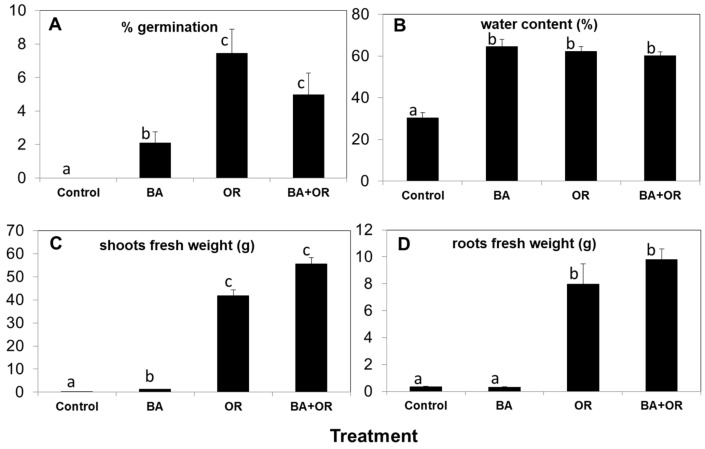
Percentage of germination (**A**), percentage of water (**B**) and fresh weight of shoots (**C**) and roots (**D**) of *C. salviifolius* plants grown for one year in unamended *gossan* (control) and in the different Technosols (mean ± standard error; *n* = 4). Significant differences between treatments are represented by different letters after Tukey’s multiple comparison tests for *p* values lower than 0.05. BA, biomass ash; OR, organic residue.

**Figure 3:**
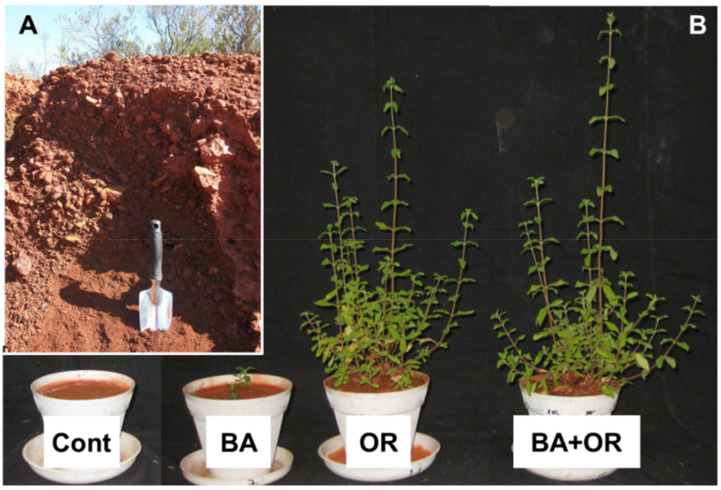
A view of the original *gossan* wastes in the vicinity of São Domingos mine (**A**), and aspect of plants grown for one year in the unamended *gossan* (Cont) and in the different Technosols after one year of growth (**B**). BA, biomass ash; OR, agriculture wastes.

Two months after sowing, the percentage of germination in the control (*gossan* wastes) was zero (Figure 2A) while seeds in Technosols containing agriculture wastes (OR and BA + OR) had the highest germination percentages. Nevertheless, at the end of the assay, there were several plants in the control treatment, which corresponded to a rate of germination of 1.13 ± 0.65 %, a rate lower than any of the other substrates at two months. Control plants were very small, with a significantly low weight of shoots and roots (Figure 2C,D), and abnormally low water contents (Figure 2B). Also, in natural environments, *gossan* material can be scarcely colonized by natural vegetation, such as *Erika australis* L., as can be seen on Figure 3A. However, these plants have very low growth rates.

The differences in shoot and root growth between the Technosols with and without agriculture wastes (OR) (Figure 2C,D) is reflected in the aspect of the plants after one year of growth (Figure 3B). Plants from the Technosol with biomass ash (BA) had very low fresh weight but showed water contents similar to the plants grown in the Technosols with OR (Figure 2B). As the low amount of plant material in the control treatment did not allow sampling for all analyses, we chose not to perform the physiological analyses in these plants, as they showed severely impaired growth and necrosis, which would render the analyses inconclusive.

### 2.4. Hydrogen Peroxide, Pigments and Antioxidants

**Figure 4:**
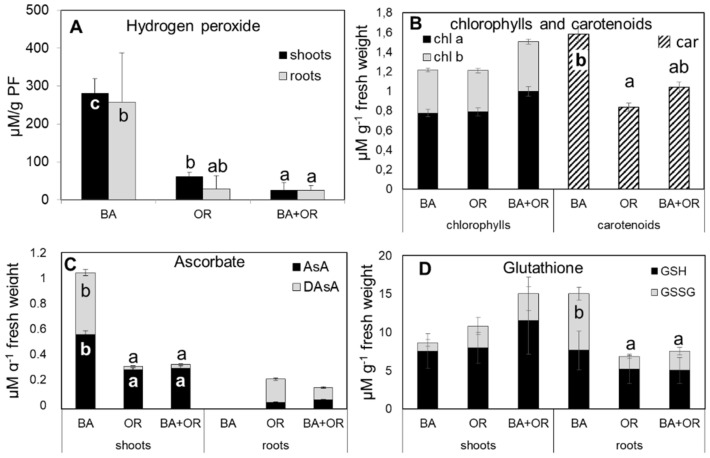
Hydrogen peroxide concentration (**A**) in shoots and roots of *C. salviifolius* plants grown in unamended *gossan* (control) and in the different Technosols; pigment (chlorophyll *a* and *b* and carotenoids) (**B**) concentration in shoots of *C. salviifolius* plants grown for one year in unamended *gossan* (control) and in the different Technosols and reduced and oxidized ascorbate (**C**) and glutathione (**D**) in shoots and roots of *C. salviifolius* plants grown in unamended *gossan* (control) and in the different Technosols. Values represent mean ± standard error for *n* = 4. BA, biomass ash; OR, agricultural wastes. Significant differences between treatments are represented by different letters after Tukey’s multiple comparison tests for *p* values lower than 0.05. When no significant differences were found, no letters were added.

**Figure 5:**
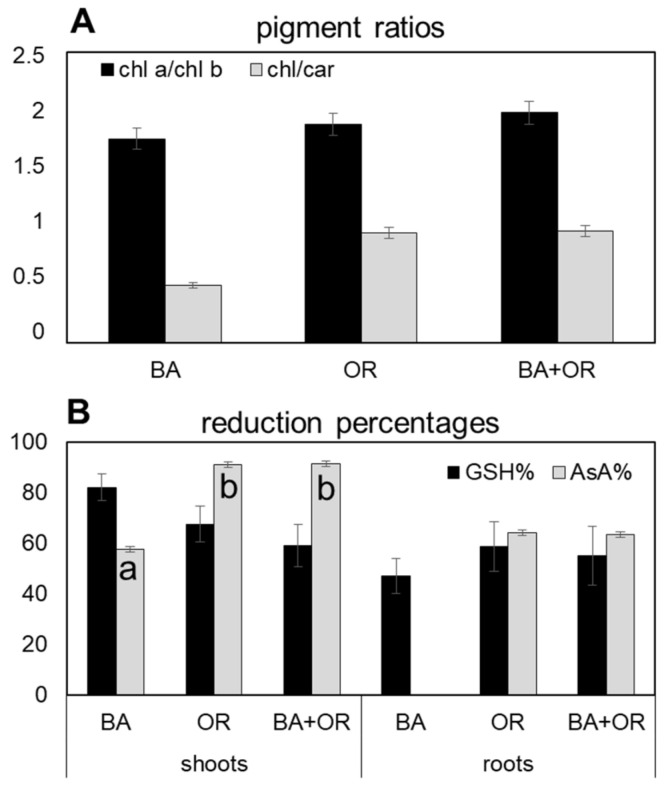
Ratio of chlorophyll *a*/chlorophyll *b* and of total chlorophyll/carotenoids (**A**) and the percentage of reduction of ascorbate and glutathione (**B**) in the shoots and in the shoots and roots, respectively, of *C. salviifolius* plants grown for one year in unamended *gossan* (control) and in the different Technosols. (mean ± standard error; n = 4). BA, biomass ash; OR, agriculture wastes. Significant differences between treatments are represented by different letters after Tukey’s multiple comparison tests for *p* values lower than 0.05. When no significant differences were found, no letters were added.

The substrate that led to the highest accumulation of H_2_O_2_ in plants was the Technosol with BA, both in shoots and roots, with values that were significantly higher than those quantified in the other two Technosols (Figure 4A). This, however, was not reflected in the pigment content (Figure 4B), with all plants showing similar values of chl *a*, chl *b* and chl *a*/chl *b* ratio (Figure 4A). The amount of carotenoids, however, responded to the higher levels of oxidative stress with significantly higher amounts in plants growing on the Technosol with BA than in the other substrates, which led to lower chl/car ratios (Figure 5A). As for ascorbate (Figure 4C), the responses to the high levels of H_2_O_2_ in BA plants were opposite in shoots and roots, with significantly higher amounts of both reduced (AsA) and oxidized (DAsA) ascorbate in shoots from the Technosol with BA than in the other treatments and with such low values in roots that they were below the detection limit. Both Technosols with organic amendments (OR and BA + OR) had plants that showed low levels of ascorbate, mostly in the reduced form (AsA), in shoots, while in roots the predominant form of ascorbate was the oxidized (DAsA). Therefore, in general, AsA% was lower in roots, but BA plants also had lower levels in shoots than the other treatments (Figure 5B). Regarding glutathione levels (Figure 4D) in roots, OR containing treatments had high levels of reduced (GSH) over oxidized (GSSH) glutathione while BA plants showed the highest total amounts and a high level of GSSG, leading to the lowest GSH% (Figure 5B). Conversely, in shoots, the highest amounts were quantified in plants from the Technosol where BA was combined with agricultural wastes (BA + OR), although these differences were not significant. 

### 2.5. Antioxidative Enzyme Activity

**Figure 6:**
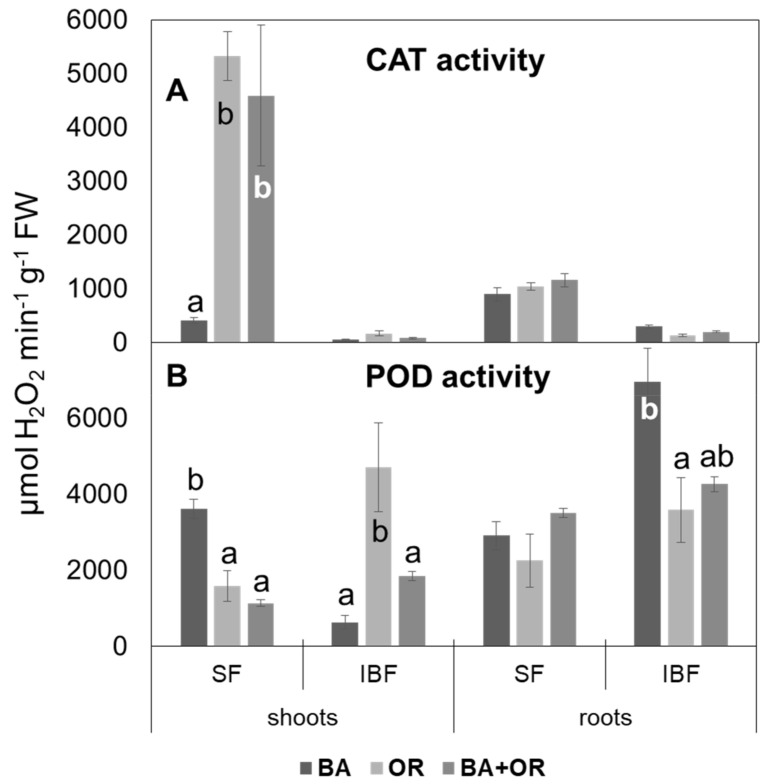
Activity of catalase (**A**) and of peroxidase (**B**) in the soluble fraction (SF) and the ionically bound fraction (IBF) of extracts of shoots and roots of *C. salviifolius* plants grown for one year in unamended *gossan* (control) and in the different Technosols. (mean ± standard error; n = 4). BA, biomass ash; OR, agriculture wastes. Significant differences between treatments are represented by different letters after Tukey’s multiple comparison tests for *p* values lower than 0.05. When no significant differences were found, no letters were added.

Catalase (CAT) activity in *C. salviifolius* was mostly located in the soluble fraction, in all treatments (Technosols and *gossan* wastes), while peroxidase activity was divided between the soluble and ionically bound fractions (Figure 6) and its proportion varied significantly with the substrate where the plants grew. Catalase activity in the soluble fraction in shoots was significantly lower in plants from Technosol with BA than in the other treatments, which showed the highest CAT activity of all tissues (Figure 6A). In roots, CAT activity did not differ between treatments, while in the ionically bound fraction, the enzyme’s activity was very low in all tissues and treatments. As for peroxidase (POD) (Figure 6B), the opposite occurred, and, in general, its activity was higher in the ionically bound fraction, especially in the roots in the Technosol with BA. In shoots, this fraction had the opposite pattern, with plants from this treatment (BA) showing the lowest values. The activity in the soluble fraction was homogenous among treatments in roots, while shoots from BA had the highest values.

## 3. Discussion

This study had the objective of evaluating the phytostabilization potential of a soil constructed (Technosol) from *gossan* materials and other wastes, that were locally available and of low cost, and of assessing the growth and physiological behavior of an autochthonous plant species well-adapted (*C. salviifolius*) to extreme environments in mine areas of the Mediterranean region. The amendments used in the Technosols were biomass ash (BA); a mixture of agriculture wastes (OR) and both (BA + OR).

*Cistus salviifolius* is a resilient species that is able to grow naturally under high total concentrations of PHE in the soil, selectively accumulating some of these elements in its tissues without showing significant symptoms of toxicity [10,11] and without severe physiological damage [18].

### 3.1. Effect of the Amendments on the Properties of the Technosols

The original *gossan* had an acid pH, low fertility and high concentrations of PHE in the total fraction; however, the concentration of these elements in the soil available fraction was, in general, lower than 8.4% of their total concentration in the soil. After *C. salviifolius* growth, the available fraction of some elements increased, indicating a potential effect of the plants on the properties of the *gossan*. In the same *gossan* material, *Lavandula pedunculata* Mill. was grown under greenhouse conditions for one year [8], but the concentrations of the elements in the available fraction were different and this increase was not observed. 

The Technosols had higher pH, EC and fertility than the *gossan* (control) (Figure 1; Table 1). After plant growth, the Technosols showed similar EC to the control, indicating a decrease of some elements in the soil solution. The C/N ratios were kept over time, and, in general, were in the same range (14–20) in all the treatments. Thus, even with higher concentrations of organic C in Technosols, the decomposition and mineralization rates were stable, allowing the maintenance of organic matter for longer periods. High C_org_ concentration ascribed to organic matter present in the Technosols with agricultural wastes (OR and BA + OR) improved the soil water-holding capacity, reflected in the higher water content of the plants grown in the Technosols containing OR, due to the capacity of organic matter to retain water and its role in soil aggregation [19]. The improvement of these physical characteristics was also observed in other Technosols made with *gossan* wastes [8,15]. This makes the plants more resilient to drought, a very important issue in the regions of the Mediterranean area, such as the Alentejo region of Portugal. The overall content of N_total_ as well as of other nutrients in the available fraction increased significantly in Technosols, especially with organic residues (OR and BA + OR), as most of the elements are bound to the organic pool and derive from the remaining nutrient solution still contained in the rockwool waste [8,15,20]. In the Technosol with a single application of BA, the concentration of Ca and Mg increased in the available fraction as a result of biomass ash application. As for the increase in N, it can be associated with a rise in microbial activity in the rhizosphere [15].

The concentration of some PHE (except Pb and Cu) in the available fraction of the Technosols with BA was, in most cases, lower after plant growth (at the end of the assay), a clear indication of the beneficial effects of the combined use of *C. salviifolius* and biomass ash that increase the pH, and, possibly, the formation of solid phases with low solubility, together with an effect of plant growth and immobilization processes that can occur in the rhizosphere and roots. For the other Technosols, the increase of Al, Ca, Fe, Mn and Zn in the available fraction is, mainly, the result of the application of rockwool and of the remaining nutrient solution that this material still retained, together with the decomposition of the other organic wastes. Similar results were obtained in other studies [8,15]. Nonetheless, there was a significant increase in the available fraction of Pb in the Technosol with BA. The effect of the application of organic compost in decreasing the availability of Pb and Cu has been documented before [21]. In the beginning of the assay, the availability of As in the Technosols increased, as observed in similar Technosols where *L. pedunculata* and *Cistus ladanifer* L. were grown [8], after one year of *C. salviifolius* growth the contrary was observed. Thus, the studied plant species can promote a decrease in the As availability.

In the present study, the incorporation of the organic residues was more effective when combined with biomass ash, since there is a combination of complementary processes associated with the environmental rehabilitation, such as the increase of pH and EC, PHE complexation with organic matter and increase of fertility (Table 2).

### 3.2. Cistus salviifolius Growth in Gossan Wastes and Technosols 

All amendments included in the Technosols’ production improved the plant vegetative development when compared with the control treatment. In fact, in the control (*gossan* wastes) the percentage of germination was zero two months after sowing, an indication of the unsuitability of this substrate for *C. salviifolius* germination and growth, as was also reported for *L. pedunculta* and *C. ladanifer* in the same *gossan* wastes. 

The application of organic matter (Technosols elaborated with OR and BA + OR) favored germination and long term plant growth as well as water content. Similar results had been reported for *Medicago sativa* L. and *C. ladanifer* in an assay that aimed at amending soils from the Rio Tinto mine (IBP, SW of Spain) [22] and in which a mixture of mine soil with compost yielded the best plant growth. This is also in accordance with previous results of *L. pedunculata* and *C. ladanifer* growth in Technosols derived from mining and agro-industrial wastes [8,15]. Furthermore, the above-mentioned beneficial effect of OR in the water-holding capacity of the soil [19] is evident in the plant’s water content and especially in their fresh weight. Also, the increase of available nutrients contributed to the stimulation of plant growth.

From the observation of Figure 1B, a possible connection between soil characteristics and translocation behavior is apparent. In general the translocation behavior was similar among treatments, except for Mn and Zn. For Mn, plants growing on substrates with low concentration of this element in the available fraction (control and Technosol with BA) translocate this element to shoots [23]. However, there is no clear explanation for the Zn storage only in roots from plants growing in Technosol with BA. 

In fact, in Technosols containing OR, *C*. *salviifolius* only accumulates P in large amounts in its shoots, while all the other elements studied were translocated to shoots in higher amounts in the unamended *gossan* wastes. Such an increase in the accumulation of elements with the increase of concentration in the media was also reported for *C. ladanifer* under hydroponic growth [24]. 

From the analysis of the values of the transfer coefficient (Table 6) and of the concentrations of PHE in plant shoots (Table 5), which are clearly below the values considered in the literature for hyperaccumulator species [25,26], it is clear that *C. salviifolius* cannot be considered a hyperaccumulator species. Therefore, it is not adequate for phytoextraction purposes, as the reusing of the elements, even with an efficient extraction technology, would not be profitable. Moreover, as the element concentrations in the available fraction of the Technosols are very small, they do not represent an environmental risk.

### 3.3. Oxidative Stress and Antioxidant Response

In Technosols containing agricultural wastes, and therefore, more organic matter, plants did not accumulate PHE to toxic amounts in shoots or in roots and thus oxidative stress, as indicated by H_2_O_2_ levels, kept low values, whereas the treatment with biomass ash induced the accumulation of this ROS to high levels. In stress caused by PHE, such as B, As, Cd and Pb it is common to observe such ROS accumulation [12,18,27,28]. In fact, in Arabidopsis, it was shown that cadmium-related H_2_O_2_ accumulation was directly linked to the oxidative stress shown by the plants [29]. Chlorophyll content in leaves did not change much with the amendments and the values quantified were in accordance with those typical for *Cistus* species [30]. In general, pigment content is affected by the excess of PHE in leaves, giving rise to typical symptoms of chlorosis which are directly related to impaired photosynthetic activity [31,32]. In the plants from the Technosol with application of BA, with high levels of oxidative stress, no changes in chlorophylls were observed, while the carotenoid content was significantly higher than in the OR treatments, an indication of an attempted response to oxidative stress. Carotenoids are an important line of plant defense against oxidative stress, as they scavenge ROS through the xanthophyll cycle, playing a significant role in the protection of the photosynthetic apparatus [33,34]. In *Cistus monspeliensis* L. under high levels of Zn toxicity, carotenoids were less sensitive to oxidative stress than chlorophylls and antocyanins, enabling them to act in ROS scavenging [30]. Another major line of defense against oxidative stress involves ascorbate and glutathione, either alone or through the ascorbate–glutathione (asc-glut) cycle [34]. Again, in the Technosol with BA amendment, plants had a different pattern of response than in the other treatments, with significantly higher levels of ascorbate with a low reduction percentage in leaves, a possible indication of an overload of the H_2_O_2_ reduction mechanism through the asc-glut cycle. In roots the levels of H_2_O_2_ were probably too high for scavenging through the asc-glut cycle and ascorbate levels were below the quantification limit, probably due to a lack of protective pigments. Glutathione, on the other hand, a stable molecule that is responsible for the scavenging of metals when in excess, kept high levels in all treatments, with high reduction rates, in roots and shoots. In fact, under metal stress conditions, glutathione levels rise, activating phytochelatin synthase to enable the formation of a phytochelatin–metal complex, with two GSH molecules that form a thiolate with a metal(loid) (such as Zn, As and Cd) [29,35,36]. This complex is then transported into the vacuole where it is stored in the form of high molecular weight complexes, the most stable and permanent storage form of metal(loid)s [35]. This seems to have been the case in the roots of plants grown in the Technosol with BA, which had very low levels of ascorbate, an indication that the asc-glut cycle was not active, but had very high levels of glutathione.

Catalase (CAT) is a peroxide scavenging enzyme with a much lower affinity for H_2_O_2_ than ascorbate peroxidase (APX) (in the order of milimolar versus micromolar for APX, [37]) and it is thus considered to be the lead ROS scavenger when H_2_O_2_ reaches high concentrations [38]. However, in shoots from the Technosol with BA, its low levels of activity must have been the cause for the accumulation of H_2_O_2_ to abnormally high rates, which impaired plant growth. In the OR containing Technosols, plants’ CAT activities were higher and this was reflected in lower ROS levels and healthy plant growth. Peroxidase activities were more evenly distributed in both fractions (soluble and ionically bound) as many of its isoforms are membrane and cell wall attached. In fact, these enzymes were particularly active in the ionically bound fraction of roots.

## 4. Materials and Methods

### 4.1. Study Area and Characteristics of the Gossan Wastes and Amendments

This study was carried out in *gossan* wastes from an abandoned mining area, São Domingos (Portuguese IPB), situated in the SE part of the IPB (±240 km S of Lisbon). This area has a typical Mediterranean climate, semiarid mesothermic with no excess water and small thermal efficiency in the hot season (Thornthwaite classification, [7]). São Domingos mine was exploited in two periods: before the Roman period for Ag, Au and Cu, and later from the middle of the 19th century until 1960 for massive sulfides and *gossan* mainly for Cu, Zn and S extraction [39]. Mining operations caused the degradation of the natural landscape including soils and superficial waters. High volumes of wastes were disposed irregularly, affecting large areas and generating acid mine drainage [2,7]. This mine waste is considered the fourth most hazardous mine waste in this mine area [40], however it is colonized naturally by tolerant and autochthonous vegetation.

Two different amendments were used to construct Technosols from *gossan* wastes. The first was biomass ash (BA) from the Huelva thermoelectric power station, applied at a concentration of 2.5 g kg^−1^ of *gossan*. The second was composed of a mixture of agriculture wastes (OR) at 120 g kg^−1^ of *gossan* in a 1:1:1 ratio containing distillation bagasse from carob fruit liqueur production, a mix of agricultural residues (substrate for strawberry cultivation together with plant remains, 3:2, *m*/*m*), and rockwool used for strawberry cultivation. The selection of these wastes is related to the fact that they can be obtained in the vicinity of the São Domingos mine, in the Huelva Thermoelectric Power Station, local distilleries, and local strawberry producers, increasing the sustainability of the *on site* treatment and keeping the CO_2_ footprint to a minimum. The third amendment consisted of a mixture of BA and OR. These three amendments were applied to *gossan* wastes in order to create Technosols that minimize mine waste hazard and create conditions favorable to the growth of *C. salviifolius,* which can also contribute to the environmental rehabilitation (phytostabilization) of the degraded area. The control consisted of *gossan* wastes. All treatments were performed in four biological replicates.

All amendments were manually mixed with the *gossan* wastes. After adding the respective amendments, soils (≈2 kg of each) were transferred to pots and allowed to incubate for 30 days at 70% of water holding capacity in the greenhouse. After this incubation, four samples of the control (*gossan* wastes) and of each Technosol were taken for characterization (Table 1).

### 4.2. Seeds and Plant Growth

The seeds of *Cistus salviifolius* L. were obtained by collecting the capsules in the mining area of São Domingos. The seeds were removed from the capsules and stored at room temperature in the dark until the moment of use.

After preparing the substrates, the sowing of *C. salviifolius* was performed, with 0.30 g of seeds in each pot (*circa* 150 seeds). Plants grew for five months, upon which time surplus plants were removed, leaving five plants per pot. These five plants left in each pot developed to complete one year of growth. At the end of the one year treatments, plants were harvested. Shoots were immediately separated from roots and washed with tap water followed by distilled water. Additionally, roots were also washed in an ultrasound bath for 30 min. *Gossan* wastes and Technosol samples from each pot were taken for analyses. Morphological and growth parameters were measured in all plants and then shoots and roots of each plant were divided in two lots, the first was frozen at −80 °C and the second was dried in an oven at 50 °C until constant weight was reached. After homogenization, *gossan* wastes and Technosol samples were sieved through a 2 mm mesh, and a part was stored at 4 °C to quantify the elements in the available fraction while the remaining sample was air-dried.

### 4.3. Gossan and Technosols Analyses

Previously air-dried *gossan*/Technosol samples were analysed for pH in water suspension (1:2.5 m/V), total organic C by wet combustion through the Walkley-Black method, extractable P and K using the Egner–Riehm method (LV ST ZM 82-97), where 0.04 mol/dm^3^ calcium lactate extraction is used as an extracting agent being acidified by hydrochloric acid up to pH 3.5–3.7, and total N by the Kjeldahl method [41].

The multi-elemental concentration of the substrates was analysed by instrumental neutron activation analysis and inductively coupled plasma (ICP) after acid digestion with perchloric, nitric, hydrochloric and hydrofluoric acids in an internationally certified laboratory ([42], ISO/IEC 17025). Quality control of the analysis is ensured by standard protocol of the certified laboratory through the use of reference materials, blanks and the realization of replicates. The reference materials used were the following: GXR-1, GXR-2, GXR-4, GXR-6, DNC-1 SDC-1, OREAS-13P and DMMAS-107. The multi-elemental concentration of the available fraction was determined by flame atomic absorption spectroscopy (all elements; F-AAS) and graphite furnace atomic absorption spectrometry (GF-AAS) after extraction in moist samples using the rhizosphere-based method proposed by Feng et al. [43].

### 4.4. Plant Chemical Analyses

The dried plant samples were homogenized and finely ground for multi-elemental chemical analysis of shoots and roots. Samples of shoots and roots were digested with ultrapure concentrated nitric acid (69%) under pressure in a microwave digester (CEM MDS 2000) at 650 W with three phases of pressure (45 Psi for 6 min, 90 Psi for 6 min and 150 Psi for 10 min) for 45 min. Samples were diluted to 10 mL with deionized water after being digested in a fume hood. The extracts obtained were analysed for total concentrations of the elements Al, As, Ca, Cu, Fe, K, Mg, Mn, Mo, Na, Pb and Zn by inductively coupled plasma mass spectrometry (ICPMS) (Thermo X Series). To test the accuracy of the method, certified reference samples of branches and leaves (NCSDC73348) and blanks were used.

### 4.5. Plant Physiological Analyses 

The physiological analyses were carried out in the frozen leaf samples (pigments) and in frozen leaf and root samples (all the other analyses). To measure pigments (chlorophylls and carotenoids) concentration, leaf samples were macerated in acetone: Tris-HCl 100 mM (80:20). The chlorophyll *a* (chl *a*), chlorophyll *b* (chl *b*), total chlorophyll (chl tot) and carotenoids (car) concentrations were assayed by spectrophotometry in a microplate reader (Sinergy HT, Biotec, Winooski, USA) at 537, 647, 663 and 470 nm, using the equations described by Sims and Gamon [44] and then expressed in µmol g^−1^ fresh weight (FW) [45].

Reduced (GSH) and oxidized (GSSG) glutathione were analysed colorimetrically in leaves and roots by the 2-vinylpiridine method described by Anderson et al. [46]. Absorbance was recorded at 412 nm in a microplate reader (Sinergy HT). Reduced and oxidized glutathione concentrations were expressed in µmol g^−1^ FW. The percentage of reduction corresponds to the percentage of GSH in the total glutathione pool and is defined as (GSH + GSSG) × 100.

Ascorbic (AsA) and dehydroascorbic (DAsA) acids were assayed using a method adapted from Okamura [47] by Carvalho and Amâncio [48]. Absorbance was recorded at 525 nm in a microplate reader (Sinergy HT). Standard curves of AsA in the range of 10–60 mM were prepared in 5 % metaphosphoric acid. The concentration of DAsA was calculated by subtracting the AsA concentration measured from the total ascorbate assayed.

Hydrogen peroxide (H_2_O_2_) production was detected using a fluorometric horseradish peroxidase (HRP) linked assay (Amplex Red assay kit, Invitrogen). Leaf material (0.1 g) was ground over activated charcoal in the presence of liquid nitrogen as described by Creissen et al. [49]. Absorbance was measured with a microplate reader (Sinergy HT) at 570 nm. Hydrogen peroxide concentrations were expressed in µmol g^−1^ FW.

### 4.6. Quantification of Enzyme Activities

Sequential protein extractions of the soluble fraction and of the ionically bound fraction were performed and enzyme activities were made in triplicate on each of the fractions, in leaves and roots of the plants, according to the method described by Pang et al. [50] and Ingham et al. [51] and adapted to *C. ladanifer* by Santos et al. [52]. Catalase (CAT) activity was measured according to the method described by Wong and Whitaker [53] and Chance and Maehly [54] while peroxidase (POD) activity was performed according to the guaiacol method of Yuan and Jiang [55] and Chance and Maehly [54].

### 4.7. Statistical Analyses

Averages and standard deviations were obtained in the sets of data of *gossan*/Technosols and plants from control and Technosols. Statistical analyses were performed by the statistical software SPSS version 18.0 for Windows. The data were analysed by a one way ANOVA followed by Tukey’s test (*p* < 0.05). Quality control of the elemental analysis of soils and plants was made with laboratory standards, as described above. All the physiological analyses were performed in technical triplicates.

The translocation factor from roots to shoots (TF = [total shoots element]/[total roots element]) was also calculated [10,11].

## 5. Conclusions

The application of amendments, especially those containing organic matter (mixture or single application), to the *gossan* wastes together with plant growth (*C. salviifolius*), using a phytostabilization approach, allowed for the improvement of the soil–plant system. Under those conditions, there was an improvement of the physical and chemical properties of the mine waste, namely the decrease in the concentration of the majority of PHE in the available fraction and the increase of fertility. The structure was also improved in the Technosols containing agriculture wastes. As a whole, a much faster environmental rehabilitation was observed in the Technosols. 

Plants in the Technosols containing agricultural wastes with/without biomass ash (OR and BA + OR) grew larger, showed lower levels of oxidative stress and a better functioning of stress responses. These plants also accumulated lower amounts of PHE (e.g., As, Cu, Pb and Zn) in their shoots (TF < 1), as well as in the roots and can be classified as tolerant plants. The mechanisms involved in this tolerance range from the functioning of the ascorbate-glutathione cycle to detoxify ROS in the less stressful conditions (Technosols with OR) to the focus on metal(loid) detoxification through phytochelatins in roots of the most stressful condition (Technosol with BA). *Cistus salviifolius* growing in Technosols, even in conditions where the mine waste is the main component, shows high potential to accelerate the rehabilitation processes of areas with heavy PHE contamination. In these conditions, the physical and chemical properties of the mine wastes can be significantly improved with suitable cost-effective amendments. 

## Figures and Tables

**Table 1:** Chemical elements’(CE) mass fraction [*w*/(g/kg)] in the total fraction of the *gossan* wastes and organic/inorganic wastes used as amendments (adapted from [8]). Biomass ash was obtained from the Huelva thermoelectric power station; agriculture residues are comprised of substrate for strawberry cultivation and plant remains in a ratio of 3:2 (*m/m*); carob waste is the distillation bagasse from carob fruit liqueur production; and rockwool waste is rockwool used for strawberry cultivation. Values for *gossan* are the mean ± standard error for *n* = 4. The other values are of composite samples, representative of each sampled material.

CE	*Gossan* Waste *w*/(g/kg)	Agriculture Waste *w*/(g/kg)	Carob Waste *w*/(g/kg)	Rockwool Waste *w*/(g/kg)	Biomass Ash *w*/(g/kg)
Al	20.9 ± 0.9	4.06	0.6 × 10^−3^	53.4	30.9
As	10.0 ± 1.2	2.3 × 10^−3^	0.8 × 10^−3^	<0.5 × 10^−3^	0.03
Ca	0.2 ± 0.1	22.56	11.02	139.0	159.0
Cu	0.31 ± 0.0	44.6 × 10^−3^	9.4 × 10^−3^	0.08	0.22
Fe	240.0 ± 23.1	2.96	0.99	39.90	19.8
K	3.3 ± 0.1	1.495	0.96	6.80	25.4
Mg	0.3 ± 0.1	2.34	> 9.63	42.10	14.7
Mn	0.1 ± 0.1	0.25	43.1 × 10^−3^	1.85	2.88
Na	0.8 ± 0.1	0.31	0.40	13.10	5.5
Pb	33.4 ± 1.6	5.0 × 10^−3^	0.9 × 10^−3^	<3 × 10^−3^	0.10
Zn	0.11 ± 0.0	75.6 × 10^−3^	18.5 × 10^−3^	0.20	0.25

**Table 2:** Physicochemical characteristics of the *gossan* waste (control) and of the Technosols composed of *gossan* wastes with biomass ash (BA), a mixture of several agricultural residues (OR) or both (BA + OR), collected at the beginning of the assay (one month after incubation and before the sowing of *Cistus salviifolius*) and after one year of *C. salviifolius* growth (mean ± standard error; *n* = 4).

	Control	Technosols		
		BA	OR	BA + OR
Beginning of the assay				
pH	3.8 ± 0.1 ^a^	5.9 ± 0.1 ^b^	6.0 ± 0.1 ^b^	6.8 ± 0.2 ^c^
CE (µS/cm)	137 ± 20.5 ^a^	240 ± 13 ^b^	620 ± 293 ^c^	529 ± 34.2 ^c^
C_org_ (g/kg)	2.2 ± 0.2 ^a^	2.6 ± 0.5 ^a^	9.0 ± 1.7 ^c^	5.8 ± 0.4 ^b^
P_ext_ (mg/kg)	0.2 ± 0.1 ^a^	99.4 ± 189 ^ab^	547 ± 416 ^ab^	298 ± 195 ^b^
K_ext_ (mg/kg)	16.9± 0.5	122.5 ± 58.9	308.6 ± 87.9	395.3 ± 26.4
N_Total_ (mg/kg)	126 ± 12.5 ^a^	145 ± 132 ^a^	431 ± 31.5 ^c^	319 ± 24.8 ^b^
After one year of *Cistus salviifolius* growth				
pH	4.0 ± 0.1 ^a^*	5.6 ± 0.3 ^b^*	6.5 ± 0.3 ^b^*	6.9 ± 0.4 ^c^
CE (µS/cm)	114.7 ± 30.7 ^a^	114.3 ± 85.2 ^a^*	180.7 ± 113.4 ^a^*	223.2 ± 230.8 ^a^*
C_org_ (g/kg)	3.2 ± 0.9 ^a^	3.6 ± 1.1 ^a^	10.6 ± 1.6 ^b^	9.4 ± 0.4 ^b^*
P_ext_ (mg/kg)	<0.2 ^a^	1.0 ± 0.5 ^a^	622 ± 566 ^a^	930 ± 996 ^a^
K_ext_ (mg/kg)	14.1 ± 2.5	38.4 ± 4.1	101.7 ± 12.7	123.5 ± 28.2
N_Total_ (mg/kg)	188 ± 38.2 ^a^*	193 ± 67.4 ^a^	658 ± 86.9 ^b^*	638 ± 75.1 ^b^*

Different letters in the same line indicate significant differences among treatments (*p* < 0.05). * In the values obtained after one year of *Cistus salviifolius* growth indicates significant differences between that value and the one obtained at the beginning of the assay.

**Table 3:** Chemical elements (CE) mass fraction [*w*/(mg/kg)] in the available fraction of the *gossan* waste (control) and of the Technosols composed of *gossan* wastes with biomass ash (BA), a mixture of several agriculture residues) (OR) or both (BA + OR),) collected one month after incubation and before the sowing of *C. salviifolius* (mean ± standard error; *n* = 4).

CE	Control *w*/(mg/kg)	Technosols		
		BA *w*/(mg/kg)	OR *w*/(mg/kg)	BA + OR *w*/(mg/kg)
Al	12.6 ± 3.0 ^a^	81.3 ± 35.6 ^c^	63.3 ± 24.1 ^b^	132 ± 26.4 ^d^
As	0.2 ± 0.02 ^a^	4.8 ± 0.8 ^c^	3.8 ± 0.6 ^b^	4.5 ± 1.8 ^bc^
Ca	15.0 ± 6.1 ^a^	154 ± 29.8 ^b^	721.0 ± 119.0 ^c^	995.0 ± 176.0 ^d^
Cu	1.1 ± 0.3 ^d^	0.5 ± 0.01 ^b^	0.4 ± 0.01 ^a^	0.5 ± 0.01 ^c^
Fe	18.0 ± 2.1 ^a^	84.3 ± 44.0 ^c^	65.8 ± 34.1 ^b^	108.0 ± 15.0 ^bc^
K	5.5 ± 2.2 ^a^	159.0 ± 59.5 ^c^	124.0 ± 44.9 ^b^	113.0 ± 16.6 ^bc^
Mg	2.7 ± 1.7 ^a^	5.5 ± 2.4 ^a^	85.7 ± 37.2 ^b^	125.0 ± 17.8 ^b^
Mn	0.5 ± 0.2 ^a^	14.0 ± 3.6 ^c^	11.0 ± 2.7 ^b^	14.0 ± 8.6 ^bc^
Mo	0.04 ^a^	0.04 ^a^	0.03 ^a^	0.1 ± 0.04 ^b^
Na	3.5 ± 1.4 ^a^	71.3 ± 27.3 ^c^	55.6 ± 20.7 ^b^	111.0 ± 12.8 ^d^
Pb	10.4 ± 6.3 ^d^	2.2 ± 0.03 ^b^	1.8 ± 0.03 ^a^	2.3 ± 0.02 ^c^
Zn	<0.7 ^a^	4.4 ± 2.7 ^c^	3.5 ± 2.1 ^b^	1.8 ± 0.5 ^bc^

Different letters in the same line indicate significant differences among treatments (*p* < 0.05).

**Table 4:** Chemical elements (CE) mass fraction [*w*/(mg/kg)] in the available fraction of the *gossan* waste (control) and of the Technosols, composed of *gossan* wastes with biomass ash (BA), a mixture of several agriculture residues) (OR) or both (BA + OR), collected after one year of *C. salviifolius* growth (mean ± standard error; *n* = 4).

CE	Control *w*/(mg/kg)		Technosols	
		BA	OR	BA + OR
		*w*/(mg/kg)	*w*/(mg/kg)	*w*/(mg/kg)
Al	31.0 ± 1.0 ^a^*	41.2 ± 5.5 ^b^*	161 ± 77.0 ^c^	179 ± 101 ^c^
As	0.2 ± 0.02 ^a^	0.2 ± 0.1 ^a^*	1.3 ± 0.3 ^b^*	1.4 ± 0.1 ^b^*
Ca	49.0 ± 11.1 ^a^*	328.0 ± 41.3 ^b^*	1047.0 ± 317.0 ^c^	1054.0 ± 264.0 ^c^
Cu	1.7 ± 0.1 ^c^*	1.3 ± 0.2 ^b^*	<0.9 ^a^*	<0.09 ^a^*
Fe	48.7 ± 3.7 ^a^*	42.1 ± 5.6 ^a^	136.0 ± 47.7 ^b^	124.0 ± 49.1 ^b^
K	14.4 ± 2.9 ^a^*	31.3 ± 3.1 ^b^*	89.4 ± 7.5 ^c^	92.5 ± 20.9 ^c^
Mg	4.7 ± 0.4 ^a^	19.0 ± 1.2 ^b^*	7.5 ± 3.0 ^ab^*	7.3 ± 4.1 ^a^*
Mn	<0.5 ^a^*	2.7 ± 0.5 ^b^*	20.8 ± 4.6 ^c^*	17.8 ± 0.7 ^c^
Mo	<0.05 ^a^*	<0.05 ^a^*	<0.05 ^b^*	<0.05 ^b^
Na	19.3 ± 1.7 ^a^*	24.2 ± 4.0 ^a^*	150.0 ± 18.9 ^b^*	135.0 ± 42.2 ^b^
Pb	15.0 ± 4.5 ^b^	8.6 ± 4.8 ^b^*	<4.5 ^a^*	<4.5 ^a^*
Zn	1.7 ± 0.2 ^a^*	2.0 ± 0.6 ^a^	7.9 ± 4.5 ^b^	2.4 ± 0.7 ^ab^

Different letters in the same line indicate significant differences among treatments (*p* < 0.05). * indicates significant differences between the element’s concentration before (values on Table 3) and after one year of *Cistus salviifolius* growth (*p* < 0.05).

**Table 5:** Chemical elements (CE) mass fraction [*w*/(mg/kg)] in shoots and roots of *C. salviifolius*, after one year of plant growth, in *gossan* waste (control) and in the Technosols composed of *gossan* wastes with biomass ash (BA), a mixture of several agriculture residues) (OR) or both (BA + OR), (mean ± standard error; *n* = 4 except roots growing in *gossan* amended with BA, see below).

CE	Control	Technosols			
		BA	OR	BA + OR	LCT *
Shoots *w*/(mg/kg)					
Al	981.0 ± 198.0 ^c^	222.0 ± 58.3 ^b^	56.3 ± 31.3 ^a^	41.9 ± 14.5 ^a^	-
As	284.0 ± 198.0 ^c^	78.4 ± 19.4 ^b^	2.9 ± 1.1 ^a^	3.2 ± 1.0 ^a^	5–20
Ca	3969.0 ± 1164.0 ^a^	11,331.0 ± 3571.0 ^b^	4278.0 ± 724.0 ^a^	3957.0 ± 641.0 ^a^	-
Cu	116 ± 43.8 ^c^	18.0 ± 3.8 ^b^	2.5 ± 1.4 ^a^	2.1 ± 1.1 ^a^	20–100
Fe	6004.0 ± 5627.0 ^ab^	2397.0 ± 1691.0 ^a^	74.0 ± 33.7 ^a^	69.9 ± 20.4 ^a^	-
K	3772.0 ± 1558.0 ^a^	3999.0 ± 264.0 ^a^	4520.0 ± 347.0 ^a^	4115.0 ± 556.0 ^a^	-
Mg	1139.0 402.0 ^ab^	1480.0 ± 107.0 ^b^	1181.0 ± 147.0 ^a^	1162.0 ± 213.0 ^a^	-
Mn	149 ± 50.4 ^b^	647 ± 117 ^c^	81.1 ± 76.3 ^ab^	28.6 ± 3.7 ^a^	400–1000
Mo	0.5 ± 0.3 ^a^	0.7 ± 0.4 ^ab^	2.0 ± 0.4 ^b^	1.3 ± 0.5 ^b^	10–50
Na	2362.0 ± 1008.0 ^a^	1562 ± 162 ^a^	687.0 ± 134.0 ^b^	778.0 ± 226.0 ^b^	-
P	34.1± 8.0 ^a^	28.7 ± 2.3 ^a^	2177.0 ± 347.0 ^b^	1483.0 ± 293.0 ^b^	-
Pb	590.0 ± 73.4 ^c^	222.0 ± 63.9 ^b^	4.0 ± 2.9 ^a^	7.3 ± 2.7 ^a^	30–300
Zn	399.0 ± 76.4 ^c^	115.0 ± 16.6 ^b^	91.8 ± 46.1 ^ab^	43.8 ± 19.1 ^a^	100–400
Roots *w*/(mg/kg)					
Al	2960.0 ± 403.0 ^b^	3268.0 ^b^	435.0 ± 169.0 ^a^	267 ± 81.4 ^a^	
As	777.0 ± 189.0 ^c^	462.0 ^b^	123.0 ± 72.7 ^a^	64.9 ± 18.0 ^a^	
Ca	2705.0 ± 312.0 ^ab^	2847.0 ^b^	3296.0 ± 299.0 ^c^	2648.0 ± 143 ^a^	
Cu	238.0 ± 26.6 ^c^	334.0 ^b^	14.8 ± 6.4 ^a^	10.2 ± 2.7 ^a^	
Fe	14,982.0 ± 3189.0 ^c^	9873.0 ^b^	2870.0 ± 1563.0 ^a^	1470.0 ± 540.0 ^a^	
K	238.0 ± 55.8 ^a^	183.0 ^a^	1519.0 ± 262.0 ^c^	1309.0 ± 264.0 ^b^	
Mg	298.0 ± 39.7 ^a^	317.0 ^a^	772.0 ± 124.0 ^b^	759 ± 29.4 ^b^	
Mn	30.7 ± 2.5 ^a^	38.2 ^a^	206.0 ± 121.0 ^c^	116 ± 18.4 ^b^	
Mo	1.8 ± 0.8 ^ab^	1.1 ^a^	2.4 ± 0.6 ^b^	1.8 ± 0.9 ^ab^	
Na	230.0 ± 24.4 ^a^	423.0 ^b^	435 ± 125 ^b^	380 ± 139 ^b^	
P	67.0 ± 10.0 ^c^	43.7 ^b^	519 ± 58.6 ^a^	518 ± 160 ^a^	
Pb	2621 ± 441 ^c^	1984 ^b^	470 ± 269 ^a^	247 ± 67.7 ^a^	
Zn	182.3 ± 13.1 ^d^	282 ^c^	41.6 ± 15.2 ^b^	22.0 ± 2.4 ^a^	

Different letters in the same line indicate significant differences between treatments; in roots of plants growing in gossan amended with biomass ash, the element concentration correspond to a composite sample obtained from all replicates. * LCT: Values considered phytotoxic for plants in general according to Kabata-Pendias [17].

**Table 6:** Translocation factor (TF) from roots to shoots in plants of *Cistus salviifolius* growing in *gossan* wastes (control) and in the Technosols composed of *gossan* wastes with biomass ash (BA), a mixture of several agricultural residues) (OR) or both (BA + OR) (mean ± standard error; *n* = 4 except roots growing in *gossan* amended with biomass ash, BA).

	Control	Technosols		
		BA	OR	BA + OR
Al	0.33 ^b^	0.09 ^a^	0.14 ^a^	0.18 ^a^
As	0.34 ^b^	0.19 ^b^	0.03 ^a^	0.05 ^a^
Ca	1.48 ^a^	5.84 ^b^	1.30 ^a^	1.50 ^a^
Cu	0.49 ^c^	0.06 ^a^	0.17 ^b^	0.23 ^ab^
Fe	0.37 ^b^	0.49 ^b^	0.03 ^a^	0.05 ^ab^
K	15.66 ^c^	22.83 ^b^	3.04 ^a^	3.26 ^a^
Mg	3.76 ^b^	5.15 ^b^	1.54 ^a^	1.54 ^a^
Mn	4.83 ^b^	18.75 ^c^	0.35 ^a^	0.25 ^a^
Mo	0.37 ^b^	0.43 ^ab^	0.83 ^a^	0.94 ^ab^
Na	10.29 ^c^	4.22 ^b^	1.65 ^a^	2.60 ^ab^
Pb	0.23 ^c^	0.12 ^b^	0.01 ^a^	0.03 ^a^
Zn	2.19 ^b^	0.48 ^a^	2.15 ^b^	1.95 ^b^

Different letters in the same line indicate significant differences between treatments.

## Data Availability

Not applicable.
